# The role and mechanism of selenium in the prevention and progression of hepatocellular carcinoma

**DOI:** 10.3389/fonc.2025.1557233

**Published:** 2025-03-20

**Authors:** Qinying Luo, Xiaofang Bai, Xiaojiao Li, Chang Liu

**Affiliations:** ^1^ BioBank, The First Affiliated Hospital of Xi’an Jiaotong University, Xi’an, Shaanxi, China; ^2^ Department of Ultrasonography, The First Affiliated Hospital, Xi’an Jiaotong University, Xi’an, China; ^3^ Department of Clinical Laboratory, Gongli Hospital of Shanghai Pudong, Shanghai, China

**Keywords:** selenium, preventive effects, therapeutic effects, mechanism, hepatocellular carcinoma

## Abstract

Hepatocellular carcinoma (HCC) represents the most prevalent form of liver cancer. Despite notable advancements in therapeutic strategies, HCC continues to pose significant public health challenges due to its rising incidence and high mortality rates worldwide. Selenium is an essential trace element that playing a critical role in human health. Recent studies have highlighted its potential preventive and therapeutic benefits in the context of HCC. However, some *in vitro* and *in vivo* investigations have yielded inconsistent results, and the mechanisms by which selenium influences HCC are still not completely clear. This review begins by providing an extensive evaluation of the effects and mechanisms of selenium on the primary risk factors associated with HCC, including viral infections, metabolic abnormalities, and lifestyle factors. Subsequently, we outline the roles and mechanisms by which selenium influences the proliferation, metastasis, and immune microenvironment of HCC. Finally, we emphasize the imperative for further investigation into the optimal dosage and forms of selenium, as well as its effects on the HCC microenvironment, to inform the development of effective clinical strategies. This review thus provides a foundational framework for the potential clinical application of selenium in the treatment of HCC.

## 1 Introduction

Liver cancer is a major contributor to global cancer-related mortality, with projections indicating an incidence exceeding 1 million cases by 2025 (1). Hepatocellular carcinoma (HCC) is the most prevalent histological subtype of liver cancer, accounting for approximately 90% of all cases (2). The primary risk factors for HCC development include viral infections, metabolic abnormalities, and lifestyle factors (3). Despite significant advancements in therapeutic strategies, HCC survival rates remain low due to late-stage diagnosis and high rates of tumor recurrence (4), highlighting the urgent need to explore strategies aimed at preventing and mitigating the progression of HCC.

Selenium (Se), an essential trace element with critical roles in human health (5), has recently gained attention for its potential in managing HCC, particularly due to its contributions to antioxidant defense, redox signaling, DNA repair, and immune function (6). Currently, 25 selenoproteins have been identified in humans, including glutathione peroxidases (GPxs), thioredoxin reductases (TrxRs), and several other selenoproteins with yet-to-be-elucidated functions. Extensive epidemiological studies have explored the link between selenium levels and HCC risk (7).

For instance, In-Wook Kim et al. reported that serum selenium levels were significantly lower in HCC patients (67.47 *μ*g/L) compared to healthy controls (108.38 *μ*g/L) in South Korea (8), and a study by M. Buljevac et al. observed comparable findings (9). An eight-year follow-up study in Qidong County, Jiangsu Province, demonstrated that selenium supplementation reduced HCC incidence by 35.1%, with a subsequent increase in HCC rates upon withdrawal of selenium from the treatment group. Furthermore, a meta-analysis indicated that both selenium status and selenium intake were inversely related to hepatitis, cirrhosis, and HCC (10). These results emphasize the possible benefits of selenium supplementation in managing HCC. However, some *in vitro* and *in vivo* studies have yielded conflicting results regarding selenium’s impact on HCC, and the mechanisms underlying selenium’s influence on HCC remain poorly understood.

In this review, we will offer a current overview of selenium’s roles in the prevention and treatment of HCC, as well as to discuss the potential mechanisms underlying these effects. Additionally, we will suggest prospective research directions concerning the investigation of selenium in HCC. By synthesizing existing evidence, this review seeks to establish a foundation for the clinical application of selenium in the management of HCC.

## 2 The potential effects of selenium in the prevention of HCC

As mentioned above, viral infections, metabolic abnormalities, and lifestyle factors are the primary risk factors for HCC (**Table 1**). These conditions often lead to hepatitis or cirrhosis, which subsequently develop into HCC. Therefore, controlling these risk factors is crucial for reducing HCC incidence.

**Table 1:** Selenium’s preventive role in the incidence of HCC.

	Risk Factor	Selenium Functions	Reference
Viral Infections	HBV	Selenium inhibits HBV protein expression, transcription, and genome replication	(11)
		Selenium suppresses HBV replication	(12)
		Selenium can reverse HBx induced hepatotoxicity	(13)
		HBV^+^ patients exhibited significantly reduced serum selenium levels.	(12, 14, 15)
	HCV	Selenium can inhibit HCV replication.	(16)
		Selenium levels in HCV^+^ patients are notably lower than those in healthy people in clinical observations.	(16–18)
Metabolic abnormalities	Obesity	Selenium affects obesity through the regulation of reactive oxygen species (ROS).	(19, 20)
		Selenium affects obesity by modulating inflammation	(21)
		Selenium affects obesity by enhancing insulin sensitivity and improving metabolism.	(22)
	Diabetes	Selenium reduces oxidative stress and regulates diabetes.	(23, 24)
		Selenium regulates diabetes through selenium proteins.	(25, 26)
	NAFLD	Selenium can effectively alleviate hepatic oxidative stress, hepatic profibrotic responsehepatic injury, fat granule accumulation and insulin resistance during the development of NAFLD.	(27–34)
		A strong correlation is observed between serum selenium levels and the occurrence of NAFLD in clinic.	(35–37)
Lifestyle factors	Alcohol consumption	Serum selenium levels were notably reduced in patients with ALD.	(38, 39)
	Smoking	Selenium reduces the carcinogenicity of smoking through its antioxidant effects and immune regulation.	(40–42)
	Aflatoxin	Selenium controls AFB1-induced liver injury.	(43)
		Selenium reduces AFB1 toxicity by regulating mitochondrial respiration.	(44)
		Selenium can alleviate AFB1-induced cell cycle arrest.	(45)

### 2.1 Selenium reduces the occurrence of virus-induced HCC

Hepatitis B virus (HBV) and Hepatitis C virus (HCV) are the primary viruses related to HCC. They can persistently alter the host’s antiviral defenses and disrupt cellular pathways that regulate liver homeostasis, ultimately leading to viral hepatitis and HCC development (46). Selenium is the only nutrient directly correlated with viral infections. In the absence of selenium, viruses may become more prone to mutation, causing more severe damage to the organism (12, 13).

#### 2.1.1 The impact of selenium on HBV infection

**Figure 1:**
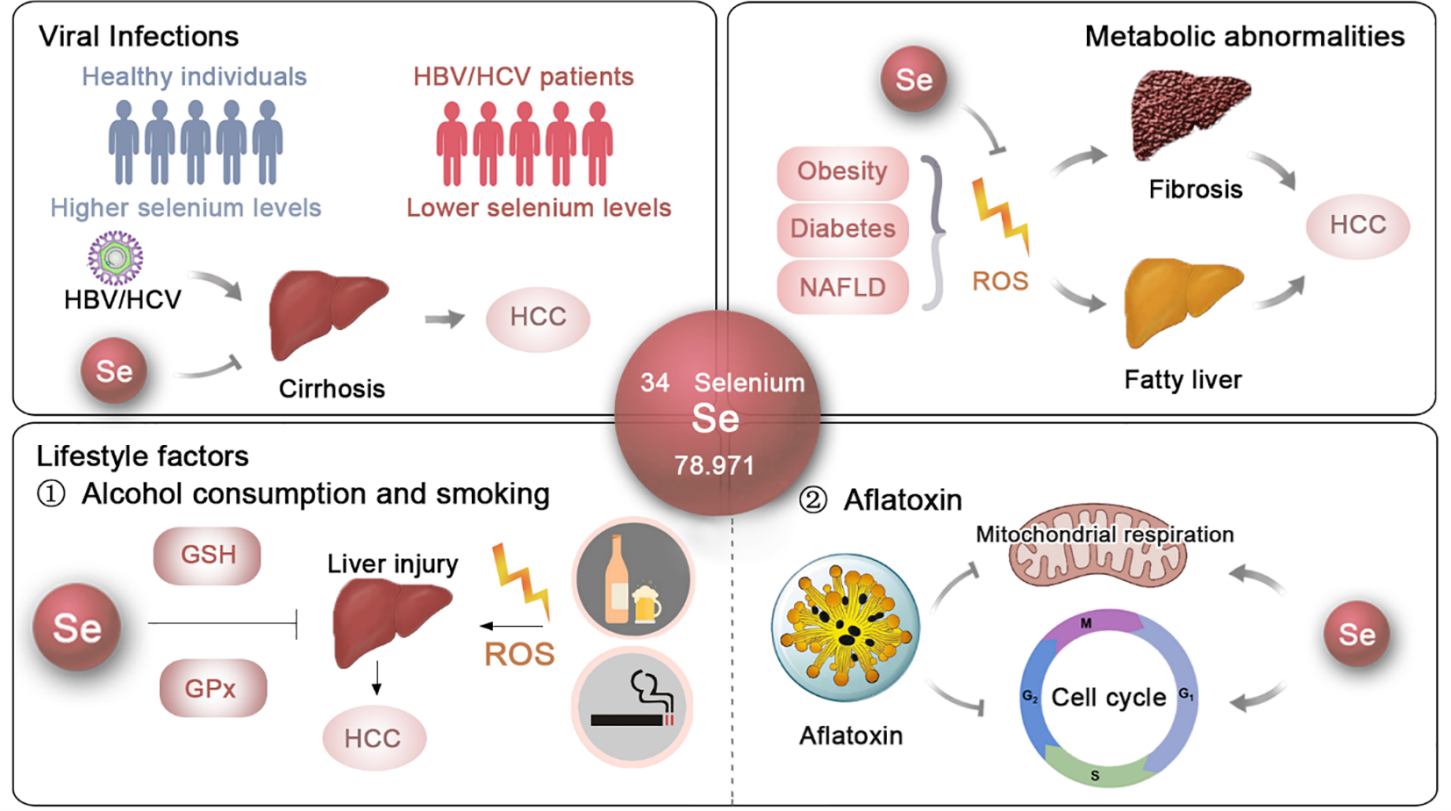
The potential roles and mechanisms of selenium in t he prevention of HCC. The effects and underlying mechanisms of selenium on the primary risk factors for HCC, including viral infections, metabolic abnormalities and lifestyle factors.

HBV infection is the cause of nearly 50% of HCC cases (47–50). The effects of selenium on HBV inhibition have been well documented. Studies have shown that sodium selenite (Na_2_SeO_3_) can suppress HBV protein expression, transcription, and genome replication in a dose- and time-dependent manner in human hepatoma cell lines Huh7 and HepG2.2.15 (11). Another study demonstrated that 5 *μ*M Na_2_SeO_3_ can suppress HBV replication by ~50% in HepG2.2.15 cells by promoting apoptotic cell death and inhibiting cellular inhibitors of apoptosis proteins (cIAPs) (**Figure 1**). Additionally, 500 nM Na_2_SeO_3_ can reverse hepatitis B virus X protein (HBx) induced hepatotoxicity by GPX4-mediated ferroptosis blockade (12). The introduction of selenoprotein P (SeP) can notably reverse HBx-induced lipid peroxidation and TNF-α upregulation in HepG2 cells (13) (**Figure 1**). These findings suggest that selenium can reduce the incidence of HBV-induced HCC.

Actually, the NHANES 2007-2018 study data indicated that individuals testing positive for hepatitis B surface antigen (HBsAg) have reduced blood selenium concentrations (14). Shi et al. confirmed that serum selenium levels are lower in HBV-positive HCC patients, and higher selenium levels are linked to better prognosis in this group (12). Similar observations were made in Pakistan by Naseem Rauf et al., emphasizing the consistent link between selenium deficiency and HBV infection (15).

#### 2.1.2 The roles of selenium in HCV infection

Regarding HCV, an *in vitro* study showed that HCV suppresses the expression of gastrointestinal glutathione peroxidase (GPx) in replicon cells, aiding viral propagation within host cells, suggesting that regulating GPx activity could provide new strategies to inhibit HCV replication (16). Clinical data also support these findings, revealing a negative correlation between HCV viral load and both selenium levels (r = -0.730) and GPx activity (r = -0.675) (17) (**Figure 1**). Additionally, Dominik Bettinger et al. found that HCV infection significantly lowers serum selenium levels, with an even greater decline in patients developing HCV-related cirrhosis (18) (**Figure 1**). In Pakistan, a significant proportion of HCV-infected patients exhibit substantially lower selenium levels compared to healthy controls (15). Overall, these findings highlight the potential effects of selenium supplementation as a therapeutic approach to reduce the risk of HBV- and HCV-induced HCC (**Figure 1**).

### 2.2 Selenium reduces the incidence of HCC caused by metabolic abnormalities

#### 2.2.1 Selenium has the potential to prevent obesity-induced HCC

Obesity is an independent risk factor or HCC (51, 52). A meta-analysis encompassing of 28 prospective cohort studies with 8,135,906 subjects revealed that an increase in body mass index (BMI) was associated with the occurrence of HCC (53). A prospective study involving over 900,000 American adults also corroborated that an increased BMI is significantly linked to heightened mortality rates due to cancer, including HCC (54).

In obese individuals, adipose tissue secretes pro-inflammatory cytokines like TNF-α and IL-6, which facilitate inflammation and tumorigenesis within the liver (55). Additionally, obesity is frequently associated with insulin resistance, which enhances the biological activity of IGF-1. A significant amount of evidence suggests that the IGF-1/IGF-1R pathway is crucial in the development of various cancers, including HCC (56). Moreover, obesity-induced gut microbiota alterations promote HCC development by generating harmful metabolites like deoxycholic acid (DCA), which trigger DNA damage and a senescence-associated secretory phenotype (SASP) in hepatic stellate cells, fostering a pro-inflammatory, tumorigenic liver microenvironment (57).

Selenium has been extensively investigated for its anti-inflammatory effects. A study demonstrated that SeNPs could influence the expression of genes associated with adipogenesis and oxidative stress in adipose tissue, suggesting a molecular mechanism through which selenium might affect obesity (58). Also, selenium supplementation has been shown to manage hyperlipidemia, by modulating oxidative stress and improving metabolic functions (59). Additionally, selenoprotein enzyme activity is crucial for maintaining the balance of pro-inflammatory and anti-inflammatory signals, which are integral components of the SASP (60). These experiments indicate that selenium may play an important role in preventing obesity-induced HCC.

A research investigation explored the correlation between fingernail selenium content and the risk of obesity among Chinese children. The findings suggested an inverse relationship, where higher selenium levels were associated with a reduced risk of obesity, indicating a potential protective role of selenium against childhood obesity (61). Similar result was highlighted in women (62). Also, a study assessing the impact of L-arginine and selenium supplementation on women with central obesity revealed that selenium markedly reduced fasting insulin levels and enhanced insulin sensitivity, thereby emphasizing its potential utility in addressing obesity-related metabolic disorders (63).

#### 2.2.2 The influence of selenium on diabetes mellitus

Diabetes mellitus has been increasingly acknowledged as a crucial risk factor influencing the onset of HCC. Numerous meta-analyses have substantiated the association between diabetes and the incidence of HCC (20, 64). A longitudinal study conducted in Taiwan, encompassing 23,820 participants monitored over a 14-year period, demonstrated that diabetes is associated with a threefold increase in the risk of developing HCC (19).

The dysregulation of glucose metabolism in diabetic individuals can also lead to the accumulation of advanced glycation end-products (AGEs), which contribute to cancer progression by inducing oxidative stress and inflammation (21). Insulin resistance is linked to heightened oxidative stress and persistent inflammation (65, 66). Additionally, high glucose levels can promote HCC metastasis through mechanisms involving metabolic enzymes like PKM2 (22).

Selenium plays a significant role in modulating diabetes, primarily by mitigating oxidative stress (59, 67). It also influences glucose metabolism by modulating phosphoinositide-3-kinase/protein kinase B (PI3K/Akt) signaling pathway, which is important for insulin action and glucose uptake. Additionally, selenium’s impact on selenoprotein P has been associated with hepatic gluconeogenesis and insulin resistance (23). Moreover, interactions between selenium levels and genetic variations in selenoprotein genes, as well as other redox-related genes, have been shown to affect diabetes risk (68). Epidemiological investigations have yielded inconsistent findings on the association between selenium levels and the likelihood of developing diabetes. Certain studies indicate that sufficient selenium intake may confer protective effects against diabetes, especially in populations with initially low selenium levels (69, 70). In contrast, other research suggests that elevated selenium exposure may be linked to an increased risk of diabetes mellitus, particularly in populations with adequate selenium status (71, 72). Thus, further research is required to determine the optimal selenium intake for diabetes prevention and management.

#### 2.2.3 The effects of selenium on non-alcoholic fatty liver disease

NAFLD is rapidly becoming the most prevalent cause of HCC (73), affecting an estimated 2 billion individuals worldwide (74). NAFLD typically starts as simple fatty liver and can progress to nonalcoholic steatohepatitis (NASH), cirrhosis, and eventually HCC (24). Although the exact mechanisms underlying NAFLD remain unclear, oxidative stress is recognized as a key factor in its development (25). Hepatic iron, which facilitates electron transfer and catalyzes the generation of reactive oxygen species (ROS), is often elevated in NAFLD patients (26). As oxidative stress increases, lipid peroxidation occurs, leading to the formation of lipid hydroperoxides. These lipid hydroperoxides, along with elevated cytokines, contribute to the direct damage of liver cell membranes and the worsening of intrahepatic inflammation. The thiol redox systems, mainly glutathione and thioredoxin, work to counteract oxidative stress within cells by reducing H_2_O_2_ and lipid hydroperoxides (75). Consequently, therapeutic strategies focusing on modulating thiol redox systems have been proposed as potential interventions for NAFLD (76) (**Figure 1**).

Selenium, a key component of thiol-containing enzymes, has been extensively investigated for its role in NAFLD. Research in animal models has provided significant insights into its therapeutic potential. For instance, Ozardali et al. showed that selenium supplementation in rodents with NAFLD induced by carbon tetrachloride (CCl_4_) not only restored liver enzyme activity but also reduced the number of stellate cells and alleviated fibrosis (28, 29). Similarly, Zhang et al. found that organic selenium could alleviate NAFLD in mice fed a high-fat diet and exposed to CCl_4_ by modulating the 5-hydroxytryptophan (5-HT)/bile acid (BA) enterohepatic circulation (31). Wang et al. demonstrated that adding selenium to the diet significantly decreased liver damage and insulin resistance in the progression of NAFLD by modulating the KEAP1/Nrf2 pathway, thereby counteracting oxidative stress through increased selenoprotein P production (27). Additionally, the administration of selenium-enriched yeast and Lactobacillus was shown to decrease hepatic oxidative stress and mitigate the profibrotic response in rats with CCl_4_-induced liver injury (32, 33). Moreover, selenium and zinc co-supplementation was found to reverse NAFLD progression by improving serum biochemical parameters and reducing lipid droplet accumulation and size in the livers of Sprague–Dawley rats (30). Notably, Shen et al. demonstrated that selenium nanoparticles (SeNPs) significantly reduced liver lipid accumulation caused by polystyrene microplastics (34), further highlighting the versatile protective effects of selenium in liver health (**Figure 1**).

In the field of nutritional epidemiology, previous research has highlighted a significant decrease in selenium levels among individuals with NAFLD (6). A study by the Health Management Center at Xiangya Hospital found that middle-aged and older adults with greater selenium consumption in their diet, even though it was less than the recommended dietary allowance in China, showed a higher occurrence of NAFLD in a dose-response manner (35). Furthermore, in a study conducted with 8,550 Chinese adults aged 40 and older in Shanghai, it was observed that participants with plasma selenium levels in the highest quartile (>247.4 *μ*g/L) had a 54% greater prevalence of NAFLD than those in the lowest quartile (<181.6 *μ*g/L), indicating a potential connection between elevated selenium levels and the prevalence of NAFLD (36). Additionally, a study on U.S. adults examined the relationships among serum selenium concentrations, serum alanine aminotransferase (ALT) activity, and the prevalence of NAFLD. The findings indicated a positive correlation between serum selenium levels and NAFLD prevalence when selenium concentrations surpassed 130 *μ*g/L, while no significant association was observed for levels below this threshold (77) (**Figure 1**).

### 2.3 Selenium reduces the risk of HCC associated with certain lifestyle factors

#### 2.3.1 Selenium alleviates the incidence of HCC induced by alcohol consumption

Excessive alcohol consumption leads to alcoholic liver disease (ALD), progressing sequentially from fatty liver to alcoholic steatohepatitis, cirrhosis, and ultimately HCC. Although the precise mechanisms are not fully understood, it is widely recognized that protein and lipid peroxidation, driven by free radical reactions, play a significant role in the development of ALD (78). In a cohort study of 80 male patients with alcoholic cirrhosis and 70 healthy male non-alcoholic controls, MDA levels, a known marker of lipid peroxidation, were significantly higher in those with alcoholic cirrhosis.Additionally, a negative correlation was found between MDA levels and the activities of antioxidant enzymes such as superoxide dismutase (SOD), GPx, and glutathione (GSH), indicating heightened oxidative stress and impaired antioxidant defense in ALD patients (79) (**Figure 1**). These findings strongly support the potential role of selenium in mitigating ALD (80).

In fact, a prevalence study of 99 patients with alcoholic cirrhosis and 20 healthy subjects revealed a significant depletion of serum selenium levels in ALD patients (38). Similarly, Rui M. Rua et al. reported that individuals suffering from alcohol use disorder and liver disease exhibited notably lower serum selenium concentrations, especially those diagnosed with ALD, and these levels were associated with the activity of GPx (39) (**Figure 1**). These observations suggest that selenium could be a potential therapeutic agent for ALD and a preventive measure against HCC (**Figure 1**).

#### 2.3.2 Selenium reduces the risk of HCC induced by smoking

Smoking is another factor leading to increased HCC risk. Numerous epidemiological studies have indicated a link between tobacco smoking and the development of HCC. For instance, the Singapore Chinese Health Study, demonstrated a dose-dependent relationship between the number of cigarettes smoked and the risk of HCC (81). Additionally, a meta-analysis highlighted the synergistic effect of smoking with HBV and HCV infections in increasing the risk of HCC, highlighting the need to tackle smoking cessation in conjunction with the prevention and treatment of viral hepatitis (82). Smoking is known to enhance the generation of reactive oxygen species, which can result in oxidative DNA damage, cytokine synthesis, and telomere dysfunction, contributing to liver carcinogenesis (83). Additionally smoking can alter the epigenetic landscape of liver cells, particularly in the context of HCV-related HCC (84).

Selenium can reduce oxidative stress by neutralizing ROS generated during smoking (42). Also, by enhancing immune function, selenium may help mitigate the immunosuppressive effects of smoking (40). Smokers have lower serum and plasma selenium levels compared to non-smokers (85, 86). Epidemiological research has looked into the connection between selenium levels and the likelihood of cancer in smokers. Some studies suggest that exposure to selenium might be connected to a decreased likelihood of lung and prostate cancer among those who have ever smoked (41, 87). Overall, selenium appears to play a multifaceted role in mitigating the adverse effects of smoking through its antioxidant and immune-modulating properties.

#### 2.3.3 Selenium averts the HCC-causing effects of aflatoxin exposure

Aflatoxin B1 (AFB1), a mycotoxin from Aspergillus species found in poorly stored food, has been extensively investigated for its contribution to the pathogenesis of HCC. The metabolic processing of AFB1 involves its conversion into a highly reactive epoxide derivative, AFB1-8,9-epoxide (AFBO). This epoxide is capable of forming covalent bonds with DNA, resulting in the formation of AFB1-DNA adducts, which are pivotal in the mutagenic process (88). Research has demonstrated that AFB1-DNA adducts are prevalent in regions with high levels of AFB1 exposure and are correlated with increased incidence of HCC (89). Furthermore, exposure to AFB1 combined with HBV infection significantly increases the likelihood of developing HCC (90). Besides its genotoxic effects, AFB1 has been shown to impair mitochondrial function and induce apoptosis through both intrinsic and extrinsic pathways, thereby causing oxidative stress and inflammation, which exacerbate liver damage and promote carcinogenesis (91, 92).

Research has shown that selenium can mitigate the toxic effects of AFB1 through several mechanisms. For one thing, selenium enhances antioxidant defense systems against AFB1 toxicity. It has been demonstrated to reduce AFB1-induced DNA damage and histological changes in the liver (43). For another thing, selenium supplementation can lessen the negative effects of AFB1 on liver mitochondrial respiratory chain complexes and improve the ratios of mitochondrial respiratory control (44). Additionally, in broilers, selenium supplementation has been observed to improve the cellular immune function impaired by AFB1, as evidenced by increased spleen weight and enhanced T cell subsets (93). Moreover, selenium’s involvement in cell cycle regulation has been underscored in studies where selenium supplementation prevented AFB1-induced G2/M phase arrest (45). Collectively, the protective effect of selenium is crucial in preventing the mutagenic and carcinogenic consequences of aflatoxin exposure.

## 3 The potential of selenium in inhibiting HCC progression

In addition to its role in the incidence of HCC, selenium has been shown to affect various aspects of HCC progression, including tumor growth, metastasis, and the immune microenvironment.

### 3.1 The impact of selenium and HCC growth

**Figure 2:**
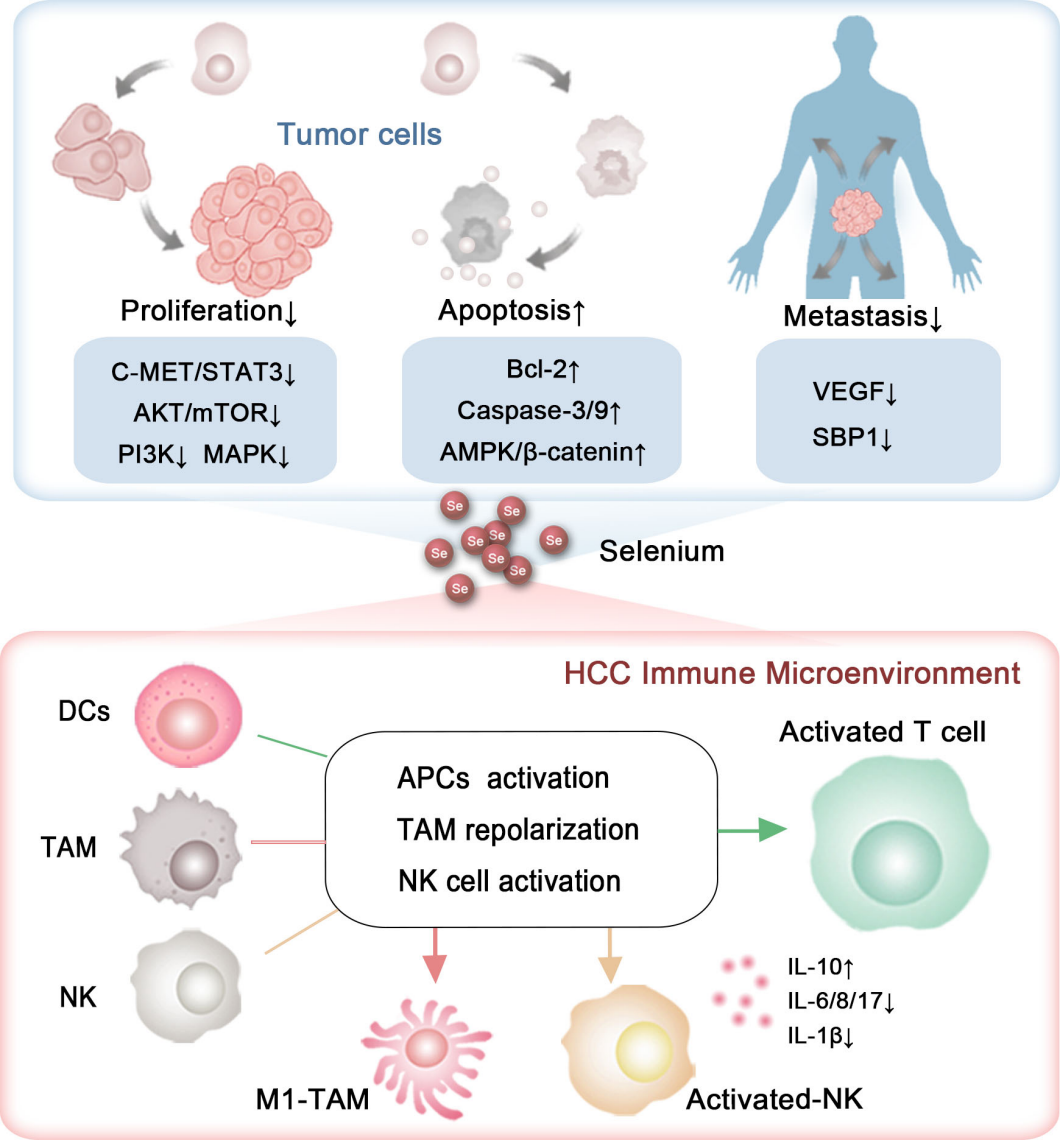
The capability of selenium to inhibit HCC progression. The roles and mechanisms by which selenium influences the growth, metastasis, and immune microenvironment of HCC.

Selenium has been shown to influence all phases of the cell cycle (94). Its anti-proliferative and pro-apoptotic effects on HCC have been extensively investigated (95) (**Figure 2**). Yang et al. reported that selenium sulfide (SeS_2_) could inhibit the C-MET/STAT3, AKT/mTOR, and MAPK signaling pathways, and trigger Bcl-2/Cyto C/Caspase-mediated intrinsic mitochondrial apoptosis both *in vitro* and *in vivo (* 96) (**Figure 2**). Additionally, in the Hep3B cell line and its xenograft tumors, selenium promoted apoptosis by activating the GSK3β-independent AMPK/β-catenin pathway (97). Cheng Wang and colleagues demonstrated that under hyperoxic conditions, H_2_Se, a product of selenium compound metabolism, is oxidized into H_2_O_2_, which blocks the Nrf2 signaling pathway, activates the MAPK signaling pathway, and enhances cell apoptosis in HepG2-bearing mice (98). A selenium containing MAPK and PI3K inhibitor (Se,Se’-1,4-phenylenebis(1,2-ethanediyl) bisisoselenourea, PBISe) has also been shown to exert chemotherapeutic effects by inhibiting the PI3K, MAPK, and STAT3 signaling pathways, leading to a significant reduction in tumor sizes in a transgenic murine HCC model (99) (**Figure 2**). Furthermore, Tohada M Al-Noshokaty and colleagues found that selenium can overcome sorafenib resistance in thioacetamide (TAA) induced HCC in rats by modulation of mTOR, NF-κB pathways and LncRNA-AF085935/GPC3 axis (100) (**Figure 2**).

In addition to selenium compounds, selenium nanocomposites, especially those incorporating additional functional components, have garnered increasing attention for their role in influencing tumor progression (101). Research by Jianshuang Jiao and colleagues revealed that selenium nanoparticles conjugated with astragalus polysaccharides induce significant morphological changes in HepG2 cells, arrest the cell cycle in the S phase and induce apoptosis through the mitochondrial route (102). Similarly, selenium nanoparticles combined with polysaccharides from Marsdenia tenacissima were shown to effectively suppress the proliferation, invasion, and metastasis of HepG2 cells, a result attributed to the triggering of the Bax/Bcl-2/Caspases and p21/Akt/Cyclin A2 signaling pathways (103). Xiao Zhang et al. claimed that selenium nanoparticles conjugated with cordyceps sinensis exopolysaccharides inhibit HepG2 cell proliferation in a manner dependent on both dose and selenium content. This inhibition is linked to disruptions in cellular membrane integrity and mitochondrial function, along with elevated levels of Bax, cytochrome c, cleaved caspase-9, cleaved caspase-3, Fas, p53, and cleaved caspase-8, and reduced levels of Bcl-2 and PARP (104) (**Figure 2**). Additionally, selenium nanoparticles decorated with polysaccharides derived from the fermented broth of Lactarius deliciosus (FLDP) demonstrated a synergistic effect in reducing toxicity and enhancing the suppression of HepG2 cells. This was achieved by promoting early apoptosis via mitochondria-mediated cytochrome C-caspase activation and ROS-induced DNA damage pathways (105).

Khaled et al. reported that berberine-loaded selenium nanoparticles exhibited a notable antitumor impact on HepG2 cells, primarily by triggering apoptosis, which was facilitated by increased expression of p53, Bax, cytosolic cytochrome C, and caspase-3 activity, along with decreased levels of Bcl-2 (106) (**Figure 2**). In a similar vein, Chi et al. demonstrated that selenium-rich royal jelly induces apoptosis in H22 tumor-bearing mice through the activation of caspase-9 and caspase-3 (107) (**Figure 2**). Moreover, selenium-enriched malt was effective in counteracting the decline in plasma glucose levels and the rise in serum calcium levels in rats with hepatoma induced by diethylnitrosamine (108). Selenium-rich amino acids were found to induce cell apoptosis in human hepatoma cells SMMC-7721 by promoting the generation of ROS (109). These studies together emphasize selenium’s potential in curbing the progression of HCC (**Figure 2**, **Table 2**).

**Table 2:** Selenium has the potential to inhibit the proliferation of HCC and promote its apoptosis.

Selenium formation	Research model	Related signaling pathways	Reference
SeS_2_	Hep3B cells Huh‐7 cells	C-MET/STAT3, AKT/mTOR MAPK signaling pathways↓	(96)
selenium	Hep3B cell line and its xenograft tumors	GSK3β-AMPK/β-catenin pathway↓	(97)
Na_2_SeO_3_ CysSeSeCys	HepG2 cells and HepG2-bearing mice under hyperoxic treatment	Nrf2 signaling↓; MAPK signaling↑	(94, 98, 108)
PBISe	Skhep1,HepG2, C3A and Huh7 cell lines	PI3K, MAPK, STAT3 signaling pathways↓	(99)
SeNPs	TAA-induced HCC in rats	mTOR, NF-κB pathway↓LncRNA-AF085935/GPC3 axis↓	(100)
Nano-complex of selenium/polysaccharide	HepG2 cells	Mitochondrial pathway Bax/Bcl-2/Caspases and p21/Akt/Cyclin A2 signaling pathways↑ Bax, Cytochrome c, cleaved caspase-9, cleaved caspase-3, Fas, p53, and cleaved caspase-8↑	(95, 101–105)
Berberine-loaded selenium nanoparticles	HepG2 cells	p53, Bax, cytosolic cytochrome C levels, and caspase-3 activity↑Bcl-2↓	(106)
Selenium-rich food	H22 tumor-bearing mice Diethylnitrosamine-induced HCC rats SMMC-7721 cells	Caspase-3, Caspase-9↑ Apoptosis↑	(107–109)

### 3.2 Selenium inhibits the metastasis of HCC through multiple mechanisms

The metastatic capability of liver cancer cells presents a significant challenge, greatly affecting the unfavorable prognosis of HCC. Research by Cheng Huang and colleagues has revealed a reduction in the expression of selenium-binding protein 1 (SBP1), a key protein involved in selenium metabolism, enhances the invasive characteristics of HCC. Patients with reduced SBP1 levels had shorter overall survival and increased disease recurrence rates (110, 111) (**Figure 2**). These results indicate that selenium supplementation could be pivotal in mitigating the metastatic progression of HCC.

To date, many studies have explored the impact of selenium on the migration and invasion of HCC. For instance, Yu Xia and colleagues discovered that selenium nanoparticles loaded with anisomycin significantly inhibit the invasive and migratory capabilities of HepG2 cells (112). Additionally, tumor metastasis relies on the development of new blood vessels, a process known as angiogenesis, which selenium has been shown to suppress by modulating the expression of vascular endothelial growth factor (VEGF). Nataliya Rohr-Udilova et al. observed that selenium treatment in a rat model of HCC enhanced hepatic GPx4 expression while decreasing VEGF (**Figure 2**). Further analysis confirmed that selenium levels had an inverse relationship with VEGF, IL-8, and the size of tumors in HCC patients (113) (**Figure 2**). Also, Fabiola Rusolo et al. reported that increasing selenite concentrations in HepG2 and Huh7 cells led to reduced levels of VEGF and three pro-inflammatory cytokines: IL-6, IL-8, and IL-17 (114) (**Figure 2**). Jia-Guo Liu et al. demonstrated that selenium-enriched malt inhibited HCC angiogenesis in rats, partly through the downregulation of VEGF and interactions with key factors such as insulin-like growth factor II (IGF-II), tumor necrosis factor-alpha (TNF-α), nitric oxide (NO), and tumor-associated nitric oxide synthase (T-NOS) (115, 116) (**Figure 2**). These findings were supported by a cohort of 29 HCC patients, where selenium levels had an inverse correlation with VEGF and IL-8 (113). The results emphasize selenium’s promise as a therapeutic option to prevent HCC metastasis (**Table 3**).

**Table 3:** Selenium possesses the ability to inhibit HCC Metastasis.

Selenium formation	Research model	Related signaling pathways	Reference
selenium-binding protein 1 (SBP1)	HCC patients	GPX1↓	(110)
selenium-binding protein 1 (SBP1)	HCC cell lines Huh-7 and 7721 HCC patients	EMT↓, CXCR4↓, AKT signaling↓	(111)
Selenium nanoparticles	HepG2 cells	Cell cycle↓, CDK-2 and ICBP90↓, cascade caspase signaling↑	(112)
Selenium supplementation	HCC patients	VEGF, IL-8↓	(113)
SELK, SELENBP1	HepG2 and Huh7 cells	IL-6, IL-8, IL-17 and VEGF↓	(114)
selenium-enriched malt	HCC rats, HCC patients	VEGF, IGF-II, TNF-α, NO, T-NOS↓	(115, 116)

### 3.3 The impact of selenium on HCC immune microenvironment remodeling

The tumor immune microenvironment (TIM) plays a crucial role in the progression of various cancers by regulating the infiltration and function of immune cells within the tumor milieu. Recently, an increasing number of studies have emphasized the effects of selenium on immune cell activity, garnering greater attention from the scientific community.

#### 3.3.1 The influence of selenium on T cell activation

T cells are capable of directly eliminating cancer cells and are crucial in orchestrating the immune response against tumors. Selenium has been shown to enhance T cell function, including proliferation, activation, and cytotoxic activity (117, 118). A Phase I clinical trial indicated that selenite treatment effectively induced tumor shrinkage in patients with squamous cell lung carcinoma. Notably, tumor reduction was observed 4 months after the initiation of selenite therapy at a dosage of 1 mg/m². Additionally, dynamic and individual variations in sPD-L1 levels were noted, and there was considerable variability in survival rates among patients. These observations suggest that selenium may possess potential therapeutic value in cancer immunotherapy (119). In mice, selenoprotein deficiency impairs T cell maturation and their response to T cell receptor stimulation (120). Supplementation of selenium in male C57B1/6J mice increases the expression of GPx4 within T cells, thereby boosting the population of T follicular helper cells (121). Also, the nanocomplex of selenium and the polysaccharide component of Pholiota adiposa significantly elevates the levels of CD3+ CD4+ T cells and CD3+ CD8+ T cell in H22 tumor-bearing mice (122).

In human subjects, supplementing with selenium boosts GPx enzyme activity in lymphocytes, which is linked to better T cell functions, including enhanced proliferation and cytokine production (123). Yi Hu and collaborators found that γδ T cells pretreated with SeNPs showed enhanced efficacy in killing cancer cells and inhibiting tumor growth compared to untreated cells, as SeNPs significantly upregulating cytotoxicity-related molecules like NKG2D, CD16, and IFN-γ while downregulating PD-1 expression in γδ T cells (124). A study derived from generally healthy population also proposed that selenium’s immune-boosting properties in humans may, at least in part, be due to enhanced activation and proliferation of B-lymphocytes and potentially improved T-cell function (125). These findings highlight the potential of selenium to activate T cells (**Figure 2**).

#### 3.3.2 The role of selenium in NK cell functions

Natural killer (NK) cells, which play a pivotal role in the innate immune system, enable the recognition and eradication of virus-infected and tumor cells through their cytotoxic functions (126). Research has shown that selenite, an inorganic form of selenium, can increase the susceptibility of tumor cells to NK cell-mediated cytotoxicity by reducing the expression of HLA-E (127). In male C57B1/6J mice, selenium led to a substantial boost in the lytic activity of activated NK cells, which demonstrated a marked increase in intermediate affinity interleukin-2 receptors (128). In mice bearing H22 tumors, seleno-ovalbumin has been shown to enhance NK cell cytotoxicity (129). Selenium-containing nanoparticles have been demonstrated to enhance NK cell function, which can be synergistically combined with radiotherapy and chemotherapy in tumor-bearing mice (130). Additionally, selenium-based nanocomplexes have been found to reverse NK cell exhaustion by downregulating the immune checkpoint PD-L1 (131, 132). Moreover, research found that in liver cancer patients, selenium metabolism in CD8+ T cells and NK cells within tumor tissues is abnormal, characterized by increased SEPP1 and decreased SELENBP (133, 134) (**Figure 2**).

#### 3.3.3 Selenium is important for the activation and differentiation of DCs

Selenium has also been reported to enhance the immune response by modulating the function of DCs. It can increase the expression of co-stimulatory molecules on the surface of DCs, such as CD80 and CD86, which are essential for the activation of T cells (135, 136). In addition, selenium influences cytokine production by DCs, promoting the secretion of pro-inflammatory cytokines like interleukin-12 (IL-12), which is essential for fostering a Th1 immune response that is advantageous in combating viral infections and suppressing tumor growth (136).

Research by Zhepeng Sun and colleagues revealed that selenium deficiency significantly inhibits the differentiation and immune function of chicken DCs by decreasing the expression of CD11c, CD40, CD86 and MHCII, as well as altering the secretion of IL-10, IL-12p40, and IFN-γ (137). Similarly, Liangliang Zhang and his team found that selenium modulates the immune function of mouse DCs through the ROS- and SELENOK-mediated ERK, Akt, and RhoA/ROCK signaling pathways (138). In both mouse and swine models, selenium nanoadjuvants have been demonstrated to be more effective than commercial inactivated vaccines in activating DCs by engaging Toll-like receptor 4 (TLR4) signaling and regulating selenoprotein expression (139). Mohammad Hossein Yazdi et al. also revealed that administering SeNPs to mice enables DCs to generate IL-12 after interacting with tumor antigens (140). These studies collectively highlight the vital role of selenium in the differentiation and activation of DCs (**Figure 2**).

#### 3.3.4 The potential effects of selenium on macrophage remodeling

Selenium has the ability to modulate macrophage polarization towards an anti-tumor phenotype, thereby enhancing the immune response against cancer cells. Research by Jie Xu and colleagues showed that SeNPs significantly activated the Tlr4/Myd88/NF-κB signaling pathway, leading to the conversion of M2 macrophages into M1 macrophages and effectively stimulating an anti-tumor immune response in mice with the H22 tumor model (122). Similarly, Shuang Li et al. found that selenium-containing membrane components can inhibit the M2-like polarization, thereby slowing the *in vivo* growth of transplanted H22 ascites hepatoma cells (141). Moreover, selenium-enriched lactobacilli have been demonstrated to alleviate CCl_4_-induced liver damage in mice by boosting macrophage functional activity (33). Moreover, inhibition of selenoprotein synthesis in myeloid cells led to reduced macrophage migration within a protein gel matrix (142). Additionally, the absence of selenoprotein K was linked to reduced receptor-mediated Ca2+ flux in macrophages (143). These findings highlight the crucial role of selenium in macrophage remodeling.

## 4 Conclusion and outlook

This study has reviewed the role of selenium in HCC, highlighting that selenium supplementation appears to be a promising approach for preventing and mitigating the progression of HCC, based on experimental trials and epidemiological data. Selenium shows great promise in lowering risk factors associated with HCC, such as viral infections, metabolic abnormalities, and lifestyle factors, primarily due to its potent antioxidant properties (**Figure 1**). Moreover, selenium has been shown to inhibit the growth of HCC cells and promote their apoptosis by enhancing its pro-antioxidant effects and modulating pathways associated with tumor development. Selenium also holds promise in controlling cancer cell migration and invasion, thereby reducing the risk of HCC recurrence and relapse. Additionally, selenium may act as an immunostimulant, activating immune cells and counteracting immune checkpoint-mediated immunosuppression within the tumor microenvironment (**Figure 2**). Despite the encouraging findings and notable advancements, several critical issues require careful consideration, and further research is needed before selenium can be widely implemented in clinical practice.

**Figure 3:**
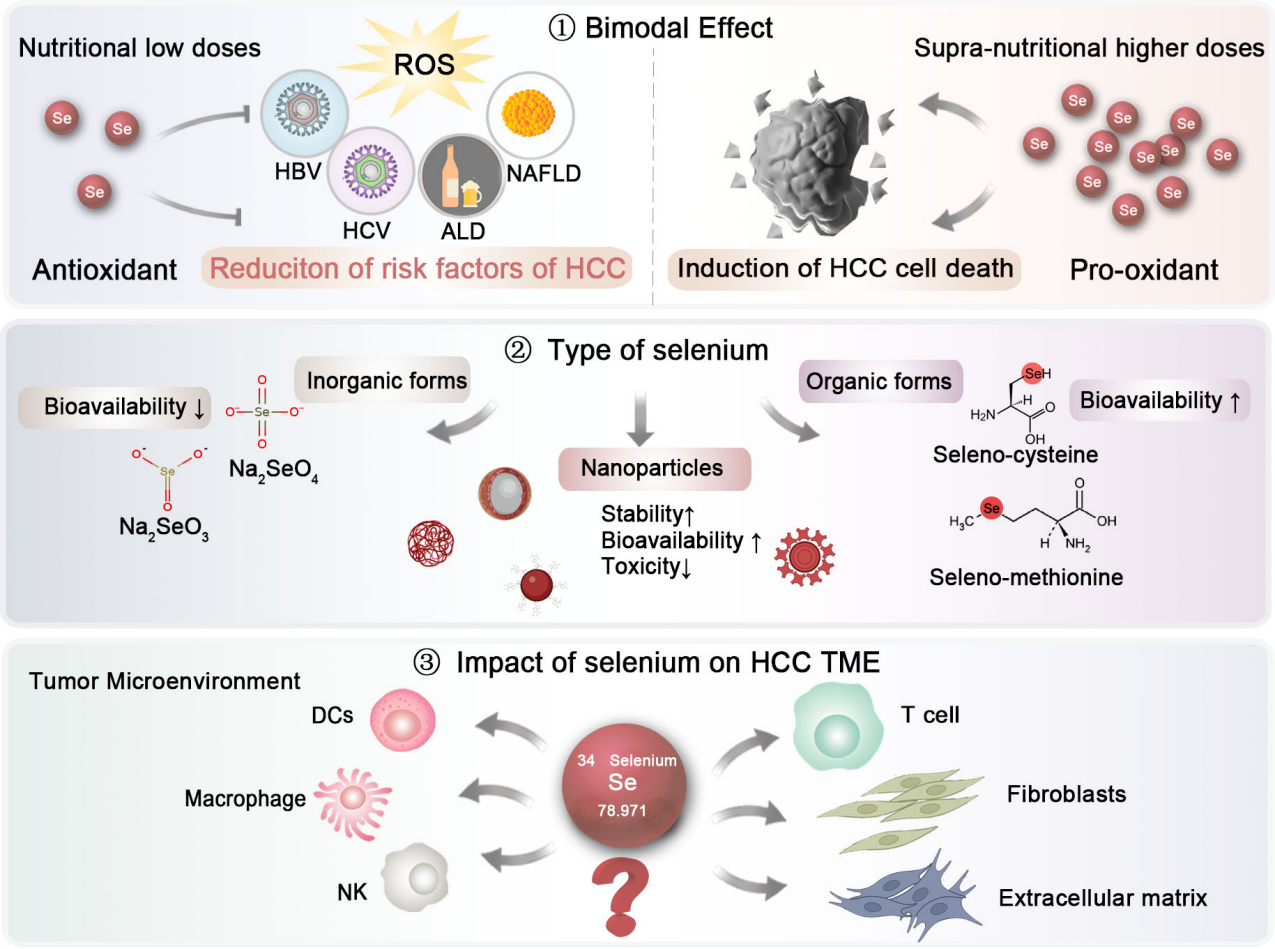
Future research directions regarding selenium investigation in HCC. The dosage and types of selenium, as well as their impact on the HCC microenvironment, should be further elucidated to establish optimal clinical application strategies.

First, the dosage is crucial (**Figure 3**). Selenium demonstrates a bimodal effect. At low, nutritional doses, selenium serves as an antioxidant, mitigating oxidative stress and thereby reducing the primary risk factors associated with HCC. In contrast, at supra-nutritional, pharmacological levels, selenium might act as a pro-oxidant, which could result in cell death in HCC. However, there is currently no consensus on the optimal reference intake levels of selenium, primarily due to the insufficient epidemiological data regarding the dose-response link between selenium and certain health effects. Given the narrow range between preventive and therapeutic doses of selenium, it is essential to conduct dose-specific preclinical studies and clinical trials to effectively utilize selenium in the prevention and treatment of HCC.

Second, the form of selenium is also of paramount importance, as it significantly influences its absorption and effects (**Figure 3**). Generally speaking, Selenium is typically absorbed from dietary sources in both inorganic and organic forms. The inorganic forms, primarily consisting of selenate (Na₂SeO₄) and sodium selenite (Na₂SeO₃), are relatively simple to synthesize and can be produced on a large scale (144). However, their bioavailability is limited, and they may exhibit genotoxic effects in the human body. In contrast, organic forms of selenium, such as selenocysteine and selenomethionine, are more readily absorbed (145). In a study involving ten groups of selenium-deficient individuals, participants were randomly assigned to receive either a placebo or daily doses of 200 or 600 *μ*g of selenium in various supplement forms, including selenomethionine, sodium selenite, or high-selenium yeast. The differences between various selenium types in their bioavailability have not been fully elucidated. In a study, ten groups of individuals lacking selenium were randomly given either a placebo or daily doses of 200 or 600 *μ*g of selenium in different supplement forms, such as selenomethionine, sodium selenite, or high-selenium yeast. Urinary excretion data revealed that selenomethionine had the highest bioavailability, while selenite showed the lowest (146). Recently, selenium nanoparticles have emerged as a novel supplementation approach, encapsulating zero-valent selenium within nano-carriers like proteins and polysaccharides to improve stability (147). This nanoform has attracted growing interest due to its enhanced bioavailability and reduced toxicity compared to traditional inorganic and organic forms. For example, Gao and colleagues demonstrated the antioxidant properties of hollow spherical selenium nanoparticles, suggesting their potential to mitigate systemic toxicity through targeted delivery (148). Consequently, selenium nanoparticles may represent a promising trend in future selenium supplementation.

Finally, the impact of selenium on HCC microenvironment warrants further exploration (**Figure 3**). While a substantial body of literature has highlighted the effects of selenium on immune cells, more in-depth investigations are essential to uncover the underlying molecular mechanisms. Comprehensive studies are also needed to evaluate the role of selenium on other elements of the tumor microenvironment (TME), including the extracellular matrix and fibroblasts, particularly at the clinical and pharmacological stages.

In summary, although selenium exhibits promise in mitigating the occurrence and progression of HCC, additional research is required to fully elucidate its effects, mechanisms, and to develop optimal strategies for its clinical implementation.
